# Challenges in the Diagnosis of Concurrent T‐Lymphoblastic Leukemia/Lymphoma and Thymic Neuroendocrine Carcinoma: A Case Report

**DOI:** 10.1155/crip/6767219

**Published:** 2026-05-25

**Authors:** Kristen Oliveira, Mary Doan, Ying Pei, Marwan Yared, Songlin Zhang

**Affiliations:** ^1^ Department of Pathology & Immunology, Baylor College of Medicine, Houston, Texas, USA, bcm.edu

**Keywords:** immunohistochemistry, neuroendocrine carcinoma, thymic carcinoma, thymus, T-lymphoblastic leukemia/lymphoma

## Abstract

The co‐occurrence of T‐lymphoblastic leukemia/lymphoma and thymic neoplasia is a rare event that can present a diagnostic challenge. In this case report, we describe a 31‐year‐old man who was found to have thymic neuroendocrine carcinoma and T‐lymphoblastic leukemia/lymphoma 2 years after initial treatment for T‐lymphoblastic leukemia/lymphoma. The thymic neuroendocrine carcinoma was initially detected in a malignant pleural effusion, and a retrospective review of a preceding lung biopsy suggested the involvement of both tumors within one tissue sample. Immunohistochemical analysis was imperative in highlighting these two elements because despite their unique cytomorphological characteristics, a limited amount of each component was seen in the tissue biopsy. Ancillary testing is critical to the recognition of these entities in small biopsies.

## 1. Introduction

Thymic neuroendocrine carcinoma and T‐lymphoblastic leukemia/lymphoma are both rare and aggressive malignancies of the anterior mediastinum. Thymic malignancies are the most common etiology of anterior mediastinal mass, accounting for 35% of cases. However, thymic neuroendocrine carcinoma is a rare subtype that accounts for only 4% of anterior mediastinal masses and often has locoregional or distant metastases at the time of diagnosis [1]. Lymphoma is the second most common etiology of anterior mediastinal masses, constituting 25% of masses (13% Hodgkin lymphoma and 12% non‐Hodgkin lymphoma). T‐lymphoblastic leukemia/lymphoma is a non‐Hodgkin lymphoma that frequently presents with lymphadenopathy or an anterior mediastinal mass [2, 3]. The co‐occurrence of thymic neoplasms (most frequently thymomas) and T‐lymphoblastic leukemia/lymphoma has been rarely reported in the literature [4]. Diagnosing co‐occurrent thymic carcinoma and T‐lymphoblastic leukemia/lymphoma can be challenging due to the limited amount of tissue biopsied, the proportion of the two elements present, and the rarity of the disease. Here, we describe a case of thymic neuroendocrine carcinoma and T‐lymphoblastic lymphoma in a patient with previously treated T‐lymphoblastic lymphoma.

## 2. Case Presentation

**Figure 1:**
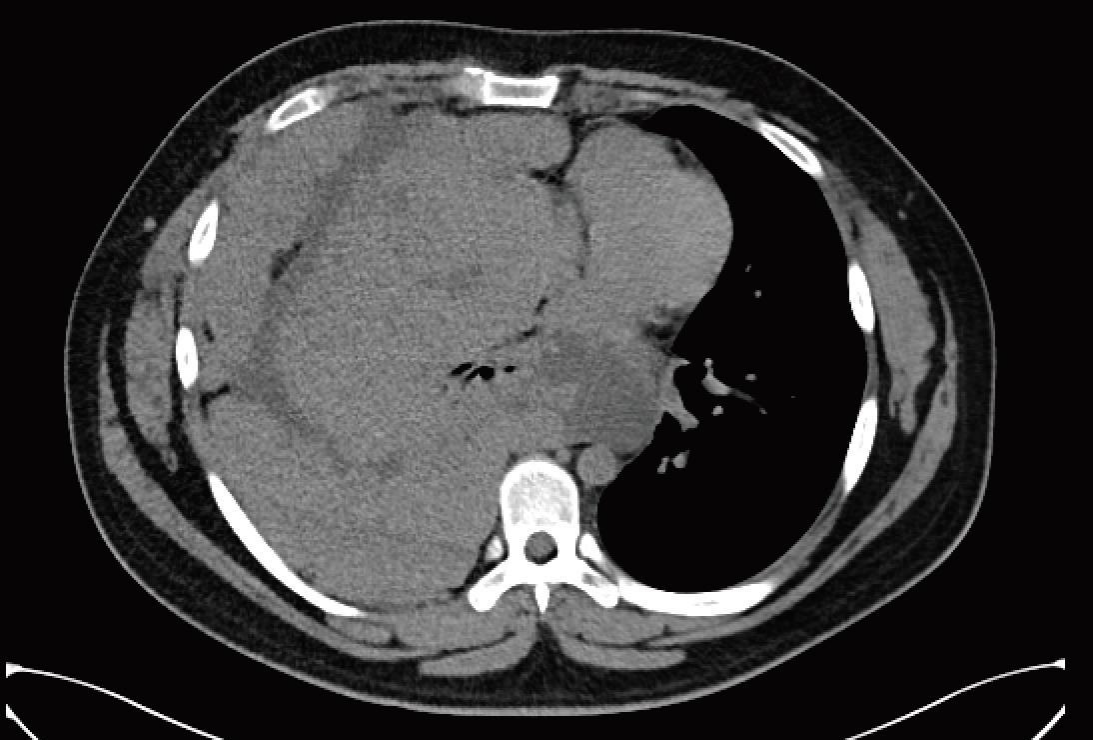
CT scan at initial diagnosis demonstrating extensive mass of the mediastinum and right hemithorax.

In May 2022, a 29‐year‐old man with no significant past medical history presented with chest pain, dyspnea, and fatigue. A CT scan showed an extensive lobulated soft tissue mass involving the mediastinum and right hemithorax with near complete collapse of the right lung, mediastinal shift, moderate right pleural effusion, and multiple subcentimeter nodules in the left lung (Figure 1). A repeat CT‐guided percutaneous biopsy of the mediastinal mass showed sheets of monotonous blastoid lymphocytic cells with admixed fibrotic stroma lacking normal lymphoid architecture. Immunohistochemistry was positive for TdT, CD3, CD10, and showed coexpression of CD4 and CD8. The cells were negative for CD34 and CD20. Flow cytometry showed increased blasts (26.8% of total cells) positive for CD1a, CD2, cCD3, CD5, CD7, CD10(dim), CD38, CD4, CD8, and TdT. The blasts were negative for B cell antigens and cMPO by flow cytometry. These results were consistent with T‐lymphoblastic lymphoma. Flow cytometry of the peripheral blood, bone marrow aspirate, and cerebrospinal fluid were negative for involvement by leukemia/lymphoma.

The patient received chemotherapy and radiation over the course of 2 years. Follow‐up PET/CT after radiation showed significant improvement in the right hilar consolidation but residual lung nodularity.

In December 2024, the now 31‐year‐old patient underwent biopsy of a right lung nodule to determine further treatment options. Pathological examination of the right lung nodule was consistent with residual T‐lymphoblastic lymphoma, showing dense lymphoid aggregates in a background of fibrosis with atypical cells positive for TdT, CD3, CD4, CD1a, and CD10 by immunohistochemistry.

**Figure 2:**
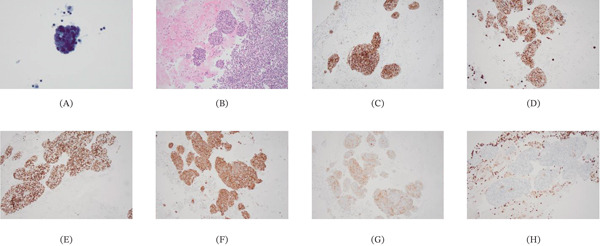
Pleural effusion cytology (Pap) showing cluster of epithelioid cells with scant cytoplasm, nuclear crowding and overlapping. (A) Cell block (H&E) from pleural effusion showing clusters of epithelioid cells and background inflammatory cells. (B) The epithelioid cells are diffusely positive for (C) MOC‐31, positive for (D) keratin and (E) p40, diffusely positive for (F) synaptophysin, (G) CD‐117, and negative for (H) CD45.

In January 2025, the patient presented with worsening chest pain and a new right pleural effusion. Cytologic analysis of the pleural fluid revealed clusters of large, atypical cells arranged in cohesive clusters. Immunohistochemical stains performed on a cell block preparation were strongly and diffusely positive for MOC‐31, calretinin, pancytokeratin, OSCAR, p40, p63, CD117, synaptophysin, CD56, and p16 with aberrant p53 staining. The cells were also focally positive for EMA, CK 5/6, GATA‐3, and chromogranin A and weakly positive for CD99. Ki‐67 was greater than 80% positive. Tumor cells were negative for CD5, CD45, CD3, CD10, TdT, CD1a, TTF‐1, PAX‐8, D2‐40, NKX3.1, CDX2, CK7, CK20, GATA‐3, SALL4, and OCT4 (Figure 2). Flow cytometry was not performed on this sample.

**Figure 3:**
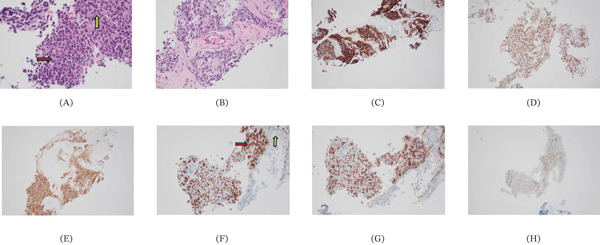
The H&E section of the lung biopsy showing malignant neoplasm with significant nuclear pleomorphism, high N/C ratio, lymphocytes (A: red arrow pointing to the small lymphocytes and yellow arrow pointing out the large carcinoma cells), and area of fibrosis and tumor necrosis (B). Tumor cells positive for (C) MOC‐31 and (D) p40, and diffusely positive for (E) synaptophysin. The lymphoid component positive for CD3 (F; red arrow pointing to the small lymphocytes and yellow arrow pointing out the large carcinoma cells), (G) CD4, and (H) TdT.

The immunohistochemical profile of the malignant cells in the patient′s pleural fluid was distinctly different from his previous T‐lymphoblastic lymphoma. The findings were most consistent with a high‐grade neuroendocrine carcinoma of thymic origin. The prior lung biopsy was reviewed due to the pleural fluid finding, and additional immunohistochemical stains were performed (Figure 3). The re‐evaluation of the right lung nodule biopsy identified a component of thymic neuroendocrine carcinoma in addition to the previously described T‐lymphoblastic lymphoma. The mediastinal biopsy performed in 2022 was also reviewed, and additional immunohistochemical stains were performed. No thymic neoplasia was identified on the initial mediastinal core biopsy.

Next generation sequencing of the pleural fluid was performed at Tempus Labs, which identified biologically relevant genomic variants including TP53 p.V197M loss of function missense variant, TET2 p.T443fs loss of function frameshift mutation, and KMT2C (MLL3) p.S229fs loss of function frameshift mutation. While TET2 loss of function is associated with dysregulation of myelopoiesis and may be more consistent with T‐lymphoblastic lymphoma, none of the variants detected are definitive markers of T‐lymphoblastic lymphoma or thymic neuroendocrine carcinoma. The initial mediastinal biopsy (2022) and the lung nodule biopsy (2024) did not have adequate tissue for further molecular studies, and these samples were unable to be compared with the pleural fluid via molecular methods.

In summary, the patient was first diagnosed with T‐lymphoblastic lymphoma from a tissue biopsy and flow cytometry of a mediastinal mass in May 2022 and received treatment. In December 2024, a lung biopsy showed T‐lymphoblastic lymphoma. One month later, pleural fluid cytology was diagnostic of thymic neuroendocrine carcinoma. This prompted a retrospective review of the prior biopsies, which identified a component of thymic neuroendocrine carcinoma in addition to T‐lymphoblastic lymphoma in the lung nodule biopsy (2024) but not in the mediastinal mass biopsy (2022).

Following diagnosis of the thymic neuroendocrine carcinoma, the patient was started on an ifosfamide, carboplatin, and etoposide regimen with the goal of treating both the carcinoma and the T‐lymphoblastic lymphoma. The patient died from complications of extensive disease with acute hypoxemic and hypercapnic respiratory failure and refractory shock 9 months after the diagnosis of thymic neuroendocrine carcinoma.

## 3. Discussion

Cases of concurrent thymic epithelial neoplasia and primary thymic T‐lymphoblastic leukemia/lymphoma have been rarely reported in the literature and most frequently involve thymoma [4]. Cases have been described of thymoma detected simultaneously [5–8], before [5, 9–11] and after T‐lymphoblastic leukemia/lymphoma [12]; the findings are summarized in Table 1. Our case is unique since none of the other case reports describe thymic neuroendocrine carcinoma and T‐lymphoblastic leukemia/lymphoma in the same tissue.

**Table 1:** Reported cases of concurrent thymic tumor and T‐lymphoblastic leukemia/lymphoma.

Authors	Year	Ref.	Age (year)/sex	Tumor type	Order of diagnosis
Macon et al.	1991	10	64 F	Invasive lymphocyte‐rich thymoma and T‐lymphoblastic leukemia/lymphoma	Thymic tumor first
Le Clef et al.	2019	9	46 M	B2 thymoma and T‐lymphoblastic leukemia/lymphoma	Thymic tumor first
Mizrahi et al.	2022	8	67 F	B1 thymoma and T‐lymphoblastic leukemia/lymphoma	Thymic tumor first
Rueda et al.	2023	4	34 M	AB thymoma and T‐lymphoblastic leukemia/lymphoma	Thymic tumor first
Rueda et al.	2023	4	48 M	B1 thymoma and T‐lymphoblastic leukemia/lymphoma	Thymic tumor first
Bendari et al.	2018	11	44 F	T‐lymphoblastic leukemia/lymphoma	T‐ALL first
Friedman et al.	1994	7	95 M	Invasive lymphocyte‐rich thymoma and T‐lymphoblastic leukemia/lymphoma	Simultaneous diagnosis
Rovera et al.	2003	6	65 M	AB thymoma and T‐lymphoblastic leukemia/lymphoma	Simultaneous diagnosis
Ito et al.	2015	5	62 M	Thymoma, thymic carcinoma, and T‐lymphoblastic leukemia/lymphoma	Simultaneous diagnosis
Rueda et al.	2023	4	43 F	B1 thymoma and T‐lymphoblastic leukemia/lymphoma	Simultaneous diagnosis

Several case reports of thymic neoplasia and T‐lymphoblastic lymphoma have elicited discussion on a possible relationship between the tumors. Mizrahi et al. proposed that untreated cancer may disrupt the anticancer surveillance function of the immune system [9]. Rueda et al. noted several cases of thymic tumor preceding lymphoma and suggested that the thymic tumor may produce oncogenic signals that promote T‐lymphoblastic leukemogenesis [5]. Weissferdt considered that treatment of a primary tumor with chemotherapy or radiation may also increase the risk of developing a secondary malignancy [4]. Alternatively, these tumors may develop independently as “collision tumors.” In our case, the initial biopsy revealed exclusively T‐lymphoblastic lymphoma, and it was only after many months of treatment and disease progression that a subsequent biopsy identified thymic neuroendocrine carcinoma in addition to persistent T‐lymphoblastic lymphoma. A potential relationship between these tumors remains unclear, and presently, there is a lack of molecular or clonal evidence supporting a shared origin.

Thymic carcinoma and T‐lymphoblastic leukemia/lymphoma have very different morphologies and immunophenotypes, and the diagnosis should not be difficult. However, recognizing the co‐occurring disease may be challenging for various reasons, including limited tissue availability. Ancillary testing can be crucial in proving the presence of a secondary thymic carcinoma after diagnosis of T‐lymphoblastic leukemia/lymphoma.

Immunohistochemical detection of keratin‐positive cells is a key diagnostic clue in differentiating thymic carcinoma from T‐lymphoblastic leukemia/lymphoma. However, the residual normal thymic epithelial cells will be positive for keratin. In this patient, diffuse positivity for pancytokeratin, OSCAR, and MOC31 in the pleural fluid sample clearly highlighted the epithelial origin of the metastatic malignancy. In similar reported cases, the NOTCH1 intracellular domain has been identified as another useful immunohistochemical marker in distinguishing thymic epithelial tumors from T lymphoblastic lymphoma due to the frequent activation of the NOTCH1 pathway in T lymphoblastic leukemia/lymphoma. In a study comparing N1ICD expression in thymomas and T lymphoblastic leukemia/lymphoma, all thymomas (*n* = 23) were negative for NOTCH1, whereas all T lymphoblastic leukemia/lymphoma cases (*n* = 16) stained positively [13].

Although flow cytometry was not performed in the 2024 lung nodule biopsy since no tissue was submitted for flow cytometry analysis, the utility of this testing has been described in similar cases to distinguish T‐lymphoblastic leukemia/lymphoma from thymic tumors. A homogenous pattern of CD4/CD8 coexpression is characteristic of T‐lymphoblastic leukemia/lymphoma, whereas a spectrum of T‐cell development is expected in benign thymocytes. Tight clustering of CD45‐dim lymphoblasts with coexpression of C10 and/or CD34 and absence of CD2 and CD5 expression are additional flow cytometric features that favor T‐lymphoblastic leukemia/lymphoma [14].

Molecular testing offers additional diagnostic utility; a monoclonal pattern of TCR‐*β* and TCR‐*γ* rearrangements on RT‐PCR and NOTCH1/FBXW7 mutations or translocations are suggestive of T‐lymphoblastic leukemia/lymphoma [9, 10]. Conversely, thymic carcinomas are molecularly heterogeneous. TP53 mutations are most frequently identified, occurring in 30%–40% of thymic carcinomas, followed by CDK2NA, KITCYLD, BAP1, and FGFR3 mutations [15, 16]. In this case, next generation sequencing identified TP53, TET2, and KMT2C (MLL3) losses. TP53 mutations have been identified in both thymic carcinomas and T‐lymphoblastic leukemia, but they are more common in thymic carcinomas (30%–40%) as compared with T‐lymphoblastic leukemia (8%) [16–18]. Loss of TET2 is commonly associated with lymphoproliferative disorders including T‐lymphoblastic leukemia, but TET2 mutations have been identified in 4%–8% of thymic carcinomas [16, 19, 20]. Additionally, KMT2C (MLL3) mutations were originally associated with pediatric mixed lineage leukemias, but have been identified in a wide range of malignancies, including thymic carcinoma [21].

In summary, the co‐occurrence of thymic neuroendocrine carcinoma and primary T‐lymphoblastic leukemia/lymphoma is rare and diagnostically challenging. Ancillary testing is critical in reaching an accurate diagnosis.

## Funding

No funding was received for this manuscript.

## Consent

No written consent has been obtained from the patient, as there is no identifiable patient information included in this report.

## Conflicts of Interest

The authors declare no conflicts of interest.

## Data Availability

The data that support the findings of this study are available on request from the corresponding author. The data are not publicly available due to privacy or ethical restrictions.
